# Inhibition of Ring-Cleaving Dioxygenases by Natural Amino Acids

**DOI:** 10.1021/acsomega.6c03268

**Published:** 2026-06-01

**Authors:** Qian Wang, Hanbin Li, Rupal Gupta

**Affiliations:** † Department of Chemistry, College of Staten Island, City University of New York, Staten Island, New York 10314, United States; ‡ Ph.D. Programs in Biochemistry and Chemistry, The Graduate Center of the City University of New York, Staten Island, New York 10016, United States

## Abstract

Inhibition of enzyme catalysis is critical for regulating cellular metabolism. Gentisate and salicylate 1,2-dioxygenases (GDO and SDO) are primary enzymes in the key gentisic pathway, which perform O_2_-dependent degradation of aromatic compounds with the aid of a ferrous cofactor. Here, we report on the inhibition of the reaction catalysis of GDO and SDO by natural amino acids. Steady-state Michaelis–Menten studies monitoring the rate of the reaction in the presence of 18 amino acids show that l-histidine primarily inhibits SDO- and GDO-mediated catalysis. In addition, l-threonine and l-asparagine also inhibit the SDO-catalyzed chemical reaction with a diminished capacity. All amino acids competitively inhibit the catalysis suggesting their binding in the catalytic cavity. While l-histidine exhibits inhibition with a binding affinity of 80 μM (SDO) and 200 μM (GDO), d-histidine does not influence the rate of the chemical reaction, demonstrating that the chirality of the amino acid is critical for supporting optimal interactions that enable binding into the catalytic pocket. Lack of inhibition by small amino acids such as l-glycine suggests that the binding of the inhibitor is mediated by both the metal-ligating backbone carboxylate group and favorable interactions between the side chain and the catalytic cavity. Using nitric oxide as a surrogate for O_2_ and EPR spectroscopy, we show that inhibitor binding does not obstruct the access of O_2_ to the active site. In addition, EPR results demonstrate that unlike substrate binding, which introduces distortions, inhibitor binding does not alter the geometry of the metal center.

## Graphical abstract

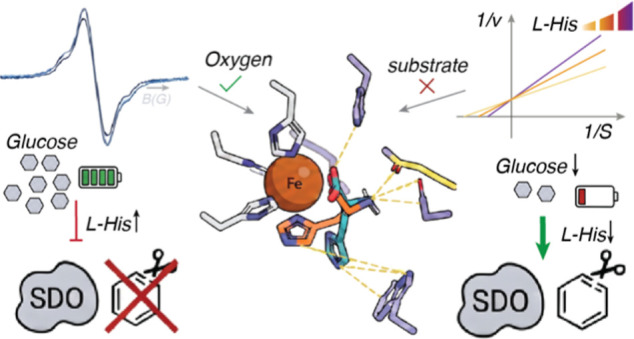

## Introduction

Aromatic ring-cleaving dioxygenases are critical for global carbon cycling. By enabling aerobic degradation of stable aromatic molecules, microorganisms utilize these compounds as their energy source and facilitate carbon transfer. C–C bond fission in an aromatic ring is a particularly challenging chemical reaction owing to the exceptional stability imparted by its delocalized electrons. However, microorganisms perform this chemical reaction at ambient conditions by recruiting oxygenases that catalyze the ring cleavage and insert oxygen atoms making these compounds accessible to other chemical processes. The majority of these oxygenases bear iron cofactors in their catalytic cavities, which facilitate the chemical reaction.^1^ In addition to this, oxygenases utilizing manganese and cobalt have also been reported.^2–4^ Aerobic degradation of aromatic compounds by microorganisms proceeds via a few established intermediates such as catechol, protocatechuate, gentisate, and hydroquinone. Gentisate is generated during the degradation of salicylate,^5^ 3-hydroxybenzoic acid,^6^ and anthranilate.^7^ Once gentisate is formed during the degradation of an aromatic compound, gentisate 1,2-dioxygenase (GDO) performs its further degradation by catalyzing its aromatic ring scission.^8–10^ Salicylate 1,2-dioxygenase (SDO) is a member of the GDO family, which was first isolated from the α proteobacterium *Pseudaminobacter salicylatoxidans* BN12.^11^ SDO can oxidize a broader spectrum of substrates, including monohydroxylated aromatic compounds such as salicylate and various substituted salicylates in addition to its primary substrate, gentisate.^11,12^

GDO and SDO are nonheme iron-dependent enzymes that insert both atoms of dioxygen into their aromatic substrates.^12,13^ Most members of the GDO family exhibit high sequence similarity.^14^ While crystal structures of substrate-free GDO from *Escherichia coli* O157:H7 and *Silicibacter pomeroyi* have been characterized,^15,16^ these structures do not provide insights into the substrate-binding mode in the catalytic cavity. In contrast, SDO has been extensively structurally characterized, and several X-ray structures with bound substrate molecules have been reported.^17–19^ These structures have demonstrated that the substrate binds to the ferrous center in a bidentate binding mode in which the neighboring hydroxyl and carboxylate functional groups of gentisate (and salicylate) ligate to the metal ion. Within the large family of oxygenases, these enzymes are classified as intradiol dioxygenases. This is because they activate the C–C double bond between the neighboring carboxylate and hydroxyl groups, leading to the scission of the bond between C1 and C2 atoms of the substrate.

Small-molecule inhibitors play a critical role in modulating uncontrolled reactions, thereby regulating various biological processes. Metabolic pathways in cells are inhibited by metabolite inhibitors that regulate enzyme activity by allosteric regulation of substrate inhibition via a feedback loop. As a key example, the glycolytic pathway utilizes glucose to generate ATP, pyruvate, and NADH. The regulation of this pathway is executed by phosphofructokinase 1 (PFK1). Upon an increase in ATP concentration, excess ATP allosterically binds to PFK 1, decreasing its efficiency and subsequently leading to the decrease in ATP production and suppression of glycolysis. This process is responsible for maintaining a stable concentration of ATP in the cells. Similarly, bacterial degradation of aromatic compounds by dioxygenases is regulated based on the substrate concentration, during which the bacteria adapt an alternate carbon source for energy generation.^20^

Given that the focal point of the chemical reaction in a metalloenzyme is the metal center, metalloenzyme inhibitors are often small molecules that coordinate to the metal ion. To achieve this, these molecules bear metal-binding groups that can ligate to the metal cofactor. In functional inhibitors, the metal-binding group is appended to the drug-like portion of the molecule via a linker.^21^ For instance, inhibitors of the aromatic ring-oxidizing tryptophan hydroxylase ligate to the iron cofactor via the metal-binding amino acid portion of the inhibitor molecule.^22^ For enzymes lacking metal cofactors, suppression of catalysis can be executed via inhibitor binding to the protein backbone, enabling the identification of a diverse set of inhibitors. In contrast, a limited subset of metal-binding inhibitors has been identified, most of which bear carboxylic acid, thiol, and phosphonate functional groups. Identification and characterization of metalloenzyme inhibitors are critical for our understanding of the biological regulation of their catalysis and the development of functional inhibitors.

Here, we investigate the regulation of the catalytic cycles of SDO and GDO by natural amino acids. Out of the 18 amino acids tested, three show clear inhibition of reaction catalysis. Steady-state Michaelis–Menten studies demonstrate that catalysis by GDO and SDO is primarily hindered by l-histidine, while l-threonine and l-asparagine also inhibit SDO-catalyzed degradation of its aromatic substrate. All amino acids competitively suppress the chemical reaction. l-Histidine shows the strongest inhibition with a binding affinity of 80 μM and 202 μM to SDO and GDO, respectively. Electron paramagnetic resonance (EPR) studies utilizing nitric oxide as a surrogate of dioxygen demonstrate that the access of O_2_ is not obstructed upon inhibitor binding to the catalytic pocket. We employed additional steady-state measurements using imidazole and d-histidine and docking studies to investigate the mode of inhibitor binding to the catalytic pocket. Our results demonstrate that in addition to the presence of the metal-binding carboxylate group, favorable interactions with the residues in the catalytic pocket play a critical role in promoting optimal inhibitor binding. Lastly, given the abundance of free amino acids in microorganisms, we postulate a feedback mechanism utilizing inhibition of GDOs by histidine to control the gentisic pathway of aromatic compound degradation while regulating cellular energy production.

## Materials and Methods

### Protein Expression and Purification

SDO cDNA sequence containing an N-terminal His-tag followed by a TEV cleavage site was cloned into vector pET-41a­(+) and transformed into the BL21­(DE3) for recombinant protein expression. The cell culture was grown at 37 °C by shaking at 220 rpm until OD_600_ reached 0.6. After that, 1 mM isopropyl β-d-1-thiogalactopyranoside and 0.1 mM ferrous ammonium sulfate were added to the growth media, and the temperature was reduced to 18 °C for 12 h. The flask vent was partially sealed to generate a nonsaturating oxygen condition. The cell culture was harvested by centrifugation at 8000 rpm and 4 °C. Upon centrifugation, the cell pellets were suspended in 50 mL of lysis buffer (50 mM Tris, 300 mM sodium chloride, 5 mM imidazole, pH 8.0) and sonicated followed by another centrifugation at 16,000 rpm for 40 min at 4 °C. The supernatant was loaded onto the HisTrap column, pre-equilibrated with loading buffer (20 mM Tris, 150 mM sodium chloride, pH 8.0). The protein was eluted with a gradient of elution buffer (20 mM Tris, 150 mM sodium chloride, 800 mM imidazole, pH 8.0) at a flow rate of 1.5 mL/min for 15 min and eluted at 240 mM imidazole gradient. Upon enzyme elution, excess imidazole was removed by a desalting column, and the buffer was exchanged to 20 mM Tris, 150 mM sodium chloride, pH 8.0. The purity of the protein was verified by the SDS-PAGE gel, and the yield of expression was estimated with a molar extinction coefficient of 74 mM^–1^ cm^–1^. 6-His tag was removed by treating the purified enzyme with TEV protease. Removal of the protease was performed by a second HisTrap column chromatography step, in which SDO was collected from the unbound fractions. Iron occupancy of the recombinant and purified enzyme was measured using protocols described previously.^23^ On average, approximately 70% of iron occupancy was observed for most protein preparations. GDO was purified using the same protocol described above.

### Steady-State Kinetic Measurements

The reaction was performed at room temperature in a reaction mixture (Tris 20 mM, sodium chloride 100 mM, pH 8.0) by observing the UV absorbance monitoring the rate of product formation upon salicylate (or gentisate) degradation at 283 nm (ε^283^ = 13.6 mM^–1^ cm^–1^).^24^ Initial velocities obtained at various substrate concentrations were fitted to the Michaelis–Menten equation to obtain the steady-state parameters, *K*_m_ and *k*_cat_. The salicylate concentration ranged from 12.5 μM to 50 μM, while the amino acids, used as inhibitors, were varied between 125 μM and 2 mM. To determine the inhibition constant *K*_i_ (herein referred to as *K*_d_), steady-state kinetic assays were performed by varying both the substrate concentration [*S*] and the inhibitor concentration [*I*]. The resulting initial velocity *v*_0_ data were globally fitted to the competitive inhibition model using Igor Pro 8. The relationship is defined by the following equation:

$$\nu =\frac{V_{\max}\cdot \left[S\right]}{K_{m}\left(1+\frac{\left[I\right]}{K_{i}}\right)+\left[S\right]}$$

### Preparation of Fe-Nitrosyl Complexes

Anaerobic protein samples (0.1 mM protein in 20 mM Tris/HCl, pH 8.0, 100 mM NaCl) were prepared by passing argon gas over the top of the sample while stirring for 30 min in a sealed vial at 4 °C. The vials were transferred into an anaerobic N_2_-filled glovebox, caps were removed, and the protein solution was allowed to equilibrate for additional 30 min. For nitrosyl complexes with substrates or inhibitors, an appropriate volume of substrate stock solutions (200–400 mM), prepared with degassed buffers in the glovebox, was added to the protein solution to generate samples with 20 equiv of substrate/inhibitor. To ensure complete binding, these samples were subsequently incubated in the glovebox for 10 min. To prepare nitrosyl complexes, the following procedure was employed. 15 mM diethylammonium salt (DEA NONOate) stock solution was prepared by dissolving preweighed NONOate in degassed 5 mM NaOH inside the glovebox. An aliquot of this stock solution, calculated based on the decay rate of DEA NONOate (half-life of 16 min at pH 7.4 and 22 °C) ensuring 5–10 equiv of NO (per Fe center), was added to each protein solution. After the addition of NONOate, each protein solution was incubated for 5 min, transferred to EPR tubes, which were then sealed before removal of the glovebox. Once the samples were taken out of the glovebox, they were immediately frozen in liquid nitrogen.

### Electron Paramagnetic Resonance Spectroscopy

EPR spectra were recorded on a Bruker Elexsys spectrometer outfitted with a dual-mode resonator and an Oxford ESR 900 cryostat. The microwave frequency, generated by a Gunn diode, was measured by a frequency counter, and the magnetic field was calibrated by an NMR gaussmeter. A modulation frequency and an amplitude of 100 kHz and 1 mTpp, respectively, were used. The temperature for all EPR measurements was calibrated using a carbon-glass resistor (LakeShore CGR-1–1000) placed at the position of the sample in an EPR tube. EPR signals quantitation was performed relative to 1 mM Cu­(II)­EDTA standard. The Cu­(II) concentration was verified by inductively coupled plasma mass spectrometry. EPR simulations for the determination of species concentration and electronic parameters were performed by the software SpinCount developed by Hendrich.^25^ The software diagonalizes the spin Hamiltonian *H* = β_e_· ***B*** · ***g*** · ***S*** + ***D*** [(***S***_*z*_^2^ – *S*(*S* + 1)/3) + *E*/*D*(***S***_*x*_^2^ – ***S***_*y*_^2^)], where all parameters have their usual definition. The simulations are least-squares fits of the experimental spectra generated with consideration of all intensity factors, which allows computation of simulated spectra for a specified sample concentration, providing quantitative determination of protein signal intensities. Concentration of the Fe-nitrosyl species derived from the simulations of all EPR spectra agreed with the expected concentration of the protein determined using the Bradford assay, based on one iron site per monomeric unit of the protein. All simulations were performed using *D* = 12 cm^–1^; σ_B_ (intrinsic line width) = 12 G and a distribution in the *E*/*D* value (σ_*E*/*D*_) = 0.15–0.2.

### Docking of Amino Acids Binding on the Enzyme Catalytic Pocket

Molecular docking simulations were performed to investigate the binding mode of the ligands with SDO using AutoDock Vina.^26^ For these calculations, the substrate-bound crystal structure of SDO (PDB: 3NJZ) was used after removing the bound substrate molecule. This PDB template was based on the following basis. The structure of SDO undergoes rearrangement upon substrate binding, and our experimental results demonstrated competitive inhibitor binding. Therefore, here we assumed that as the substrates, the inhibitors induce similar structure perturbations. Prior to docking, the protein structure was prepared using AutoDockTools (MGLTools) by removing water molecules from the PDB file, adding polar hydrogens, and assigning Kollman united atom charges. The ligands were prepared by adding Gasteiger charges and detecting rotatable bonds; nonpolar hydrogens were merged. The search space was defined as a 3D grid box centered at coordinates *x* = −9.24, *y* = −26.50, and *z* = −25.69, with dimensions of 50 × 50 × 50 Å. The exhaustiveness parameter was set to 32 to ensure adequate conformational sampling. The scoring function employed by Vina, which combines empirical and knowledge-based potentials, was used to rank the docking poses. The conformation with the lowest binding energy (highest affinity) was selected for further analysis. Interactions between the ligand and protein residues were visualized and analyzed using PyMOL. Binding affinities (BA) were calculated with AutoDock Vina (BA = *w*_1_ *E*_vdW_ + *w*_2_ *E*_Hbond_ + *w*_3Ehydrophobic_ + *w*_4Etorsion_), which were used to predict the most stable configurations with highest favorable interactions within the catalytic pocket.

## Results and Discussion

### Inhibition of the Catalytic Cycle of SDO and GDO by Select Amino Acids

We probed the inhibition of the GDO- and SDO-mediated aromatic ring fission reaction by various amino acids. For this, the influence of various amino acids on the steady-state kinetic properties was monitored. Michaelis–Menten parameters (*V*_max_, *K*_m_) were derived at pH 8.0 from the initial rates of the reactions performed at a fixed amino acid amount while varying the substrate concentration. Variation in the measured *V*_max_ and/or *K*_m_ values in the presence and absence of an amino acid is suggestive of reaction inhibition. To determine the mode of inhibition, Lineweaver–Burk plots (1/*v*_0_ vs 1/[*S*]; *v*_0_ is the initial rate and [*S*] is the substrate concentration) at various inhibitory amino acid concentrations were analyzed. In competitive inhibition where the inhibitor molecule prevents substrate binding, all 1/*v*_0_ vs 1/[*S*] lines at various inhibitor concentrations in the Lineweaver–Burk have a nonzero intercept on the 1/*v*_0_ axis at 1/*V*_max_. This mode of enzyme inhibition is distinct from uncompetitive inhibition in which all 1/*v*_0_ vs 1/[*S*] lines have an identical slope of *K*_m_/*V*_max_.

Inhibition of SDO- and GDO-mediated catalysis was monitored for 18 natural amino acids (Table 1). Out of these tested amino acids, only l-histidine inhibited the catalytic cycle of GDO. Michaelis–Menten parameters were derived for GDO-mediated oxidation of gentisate at four different histidine concentrations. All four corresponding 1/*v*_0_ vs 1/[*S*] lines in the Lineweaver–Burk plot exhibited the same nonzero intercept on the 1/*v*_0_ axis, thereby yielding identical *V*_max_ values suggestive of competitive inhibition (Figure 1a). While the *V*_max_ remains the same, the change in the slopes of the Lineweaver–Burk plots at various histidine concentrations shows a dependency of *K*_m_ on the inhibitor concentration. This dependence originates from the inhibitor competing with the substrate for binding to the catalytic cavity. Based on these measurements, histidine binds to the catalytic cavity with a *K*_d_ = 202 ± 10 μM. While the gentisate binding constant for GDO has not been reported, the measured binding affinity of histidine is comparable to the reported Michaelis–Menten constant (K_m_^gentisate^ = 130 μM).

**Table 1:** Amino Acid Inhibitors of GDO- and SDO-Mediated Chemical Reactions and Their Corresponding Binding Constants

	GDO		SDO	
amino acids	*K*_d_ [μM]	type	*K*_d_ [μM]	type
l-histidine	202	competitive	79	competitive
l-threonine	ND^a^	--	540	competitive
l-asparagine	ND^a^	--	1607	competitive
d-histidine	ND^a^	--	ND^a^	--
l-serine, l-glycine, l-proline, l-lysine, l-methionine, l-glutamine, l-leucine, l-phenylalanine, l-isoleucine, l-alanine, l-valine, l-glutamic acid, l-aspartic acid, l-cystine, l-cysteine, l-tryptophan	ND^a^	--	ND^a^	--
imidazole	ND^a^	--	ND^a^	--
l-arginine	ND^a^	--	NA^b^	--

a Weak and/or nonspecific binding detected with *K*_d_ ≫ 2 mM.

b Exhibits weak noncompetitive inhibition.

**Figure 1:**
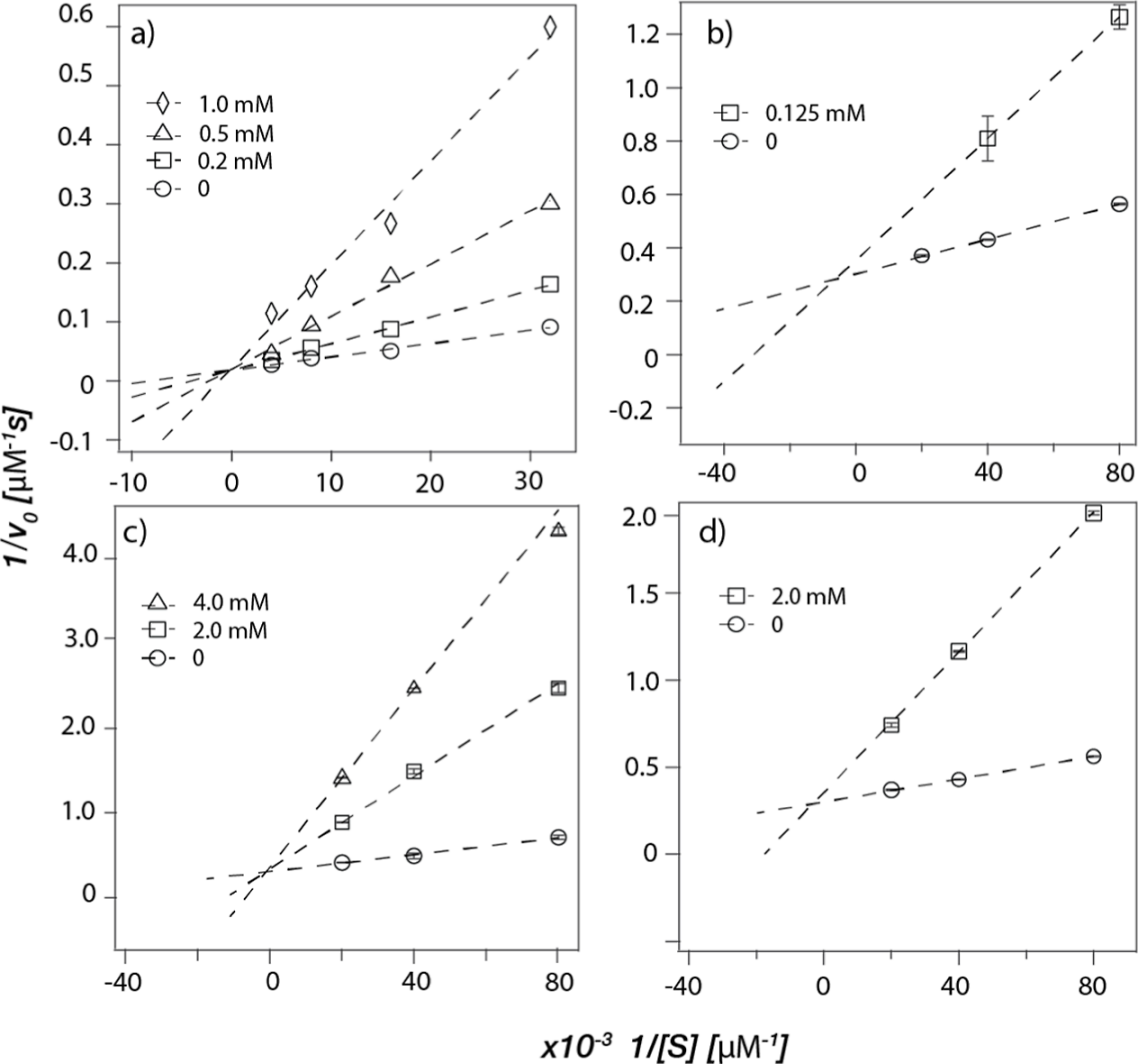
a) 1/*v*_0_ vs 1/[*S*] Lineweaver–Burk plots of GDO-catalyzed degradation of gentisate in the presence of histidine. Inhibition of SDO-mediated oxidation of salicylate observed in the Lineweaver–Burk plots for reactions in the presence of (a) histidine; (b) threonine; and (c) asparagine. See Materials and Methods for details of the experimental conditions.

Similar to GDO, SDO-catalyzed degradation of salicylate was also inhibited by histidine. However, in contrast to GDO, suppression of initial rates in the presence of threonine and asparagine suggests that the SDO-mediated reaction is also attenuated by these two amino acids. To determine the mode of inhibition and the inhibitor binding constants, steady-state kinetics experiments were independently performed under two or more inhibitor concentrations for histidine, threonine, and asparagine. For all three amino acids, the 1/*v*_0_ vs 1/[*S*] lines exhibit the same nonzero intercept on the 1/*v*_0_ axis corresponding to identical *V*_max_ in the presence or absence of the inhibitor (Figure 1b–d). These results demonstrate that all three amino acids competitively inhibit SDO-catalyzed degradation of salicylate. With competitive inhibition, these amino acids either directly bind at the substrate (or cosubstrate, i.e., O_2_)-binding site or they obstruct their access at the active site. Similar to GDO, the Lineweaver–Burk plots were used to estimate the binding affinities of each amino acid, which are presented in Table 1. Histidine exhibits the strongest competitive inhibition with a binding affinity of 80 ± 4 μM. The measured *K*_d_ of threonine was 540 ± 12 μM. While a clear inhibition effect was observed for asparagine, its binding affinity to the catalytic center was comparably weak with *K*_d_ = 1600 ± 18 μM.

### Origin of Amino Acid-Mediated Inhibition of the Chemical Reaction

Natural substrates of SDO and GDO bind to the Fe­(II) catalytic center via a bidentate mode composed of their carboxylate and hydroxyl groups. Competitive inhibition of the reaction observed in our studies could originate from the inhibitor directly binding to the metal center, thereby preventing the substrate ligation. Alternatively, the inhibitor may also suppress the reaction by binding in the catalytic pocket such that access to the substrate and/or dioxygen is obstructed. To evaluate if amino acid inhibitors obstruct dioxygen binding, we probed oxygen binding to the ferrous center with and without the inhibitor.

The first step in the catalytic cycle leading to dioxygen binding is the formation of a ferrous-oxy adduct. In most enzymes, this intermediate is short-lived and therefore rarely detected.^27–29^ A common strategy employed to probe ferrous-oxy adducts is using NO as the surrogate of O_2_. Numerous studies in the literature have demonstrated that NO binds to the O_2_-binding site at the ferrous center, resulting in the formation of a ferrous-nitrosyl complex.^30,31^ However, compared to the catalytically active Fe­(II)-O_2_ adduct, the ferrous-nitrosyl complex bears one less electron, which prevents initiation of the chemical reaction. Due to the inability of the ferrous-nitrosyl adduct to initiate the chemical reaction, once formed, these complexes are stable and amenable to detection. We have previously characterized ferrous-nitrosyl adducts of SDO and GDO in the presence and absence of various substrates using EPR spectroscopy.^23^ Upon NO binding, nitrosyl adducts of SDO and GDO show EPR signals near *g* = 4 and 2, indicative of a {Fe–NO}^7^ electronic configuration corresponding to a *S* = 3/2 spin state, which is typical for nonheme iron enzymes. Given that histidine is the primary inhibitor of GDO- and SDO-mediated catalysis, in this report, we have probed the nitrosyl complexes of these enzymes in the presence and absence of histidine. Figure 2 shows the EPR spectra of ferrous-nitrosyl adducts of SDO in the substrate-free form, in the presence of histidine, and in the presence of gentisate. The substrate-free ferrous-nitrosyl adduct (Fe­(II)-NO) exhibits near axial signals at *g* = 4.1, 4.0, and 2 (Figure 2a; only signals near *g* ∼ 4 are shown for clarity). Upon histidine binding, the Fe­(II)-His-NO complex showed signals identical to that of the Fe­(II)-NO adduct (Figure 2b). The observed EPR signals of both of these complexes can be well simulated using *S* = 3/2 and an *E*/*D* = 0.013 (Figure 2b; black trace). Additionally, simulations are in quantitative agreement with the expected amount of Fe­(II) in the sample estimated based on ferrous occupancy of the enzyme, suggesting complete NO binding. The gentisate-bound nitrosyl complex exhibits a distinct EPR spectrum compared to the substrate-free and histidine forms (Figure 2c). Upon substrate binding to the metal, the symmetry of the ferrous center is distorted as evident from the observed *g*-values at 4.15, 3.95, and 2.00. Numerical simulations, which were in quantitative agreement with the expected ferrous in the solution, suggest an increased *E*/*D* value of 0.018 for the Fe­(II)-gentisate-NO complex.

**Figure 2:**
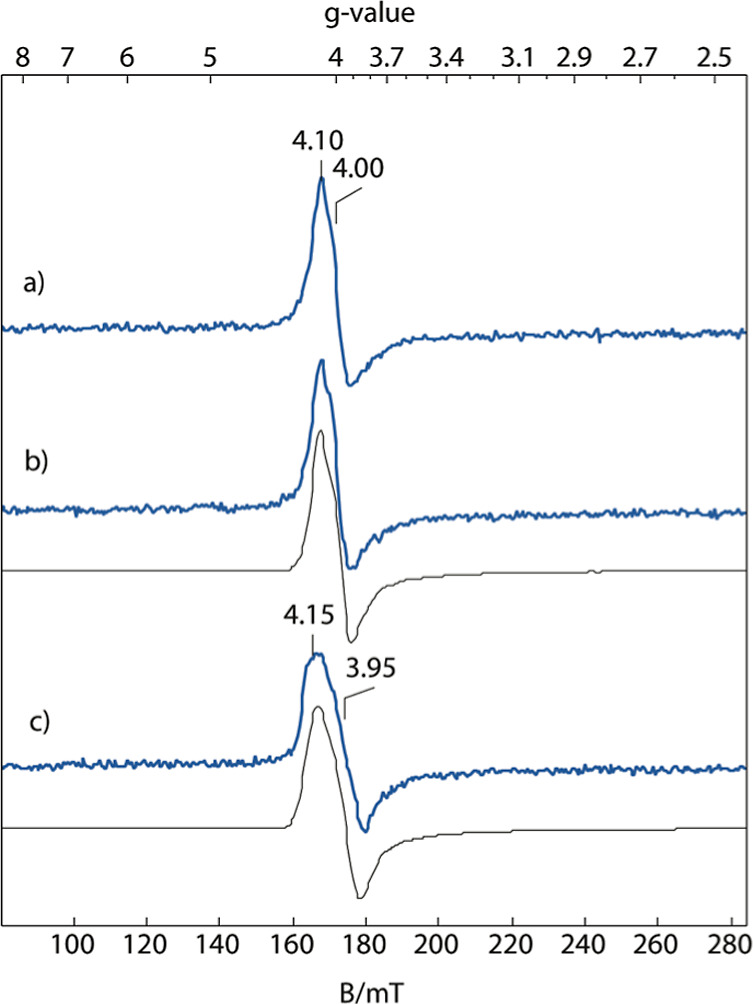
EPR spectra showing the resonance near the *g* = 4 region of the ferrous-nitrosyl complex of SDO in (a) the substrate-free form; (b) in the presence of histidine; and (c) in the presence of gentisate. Black traces show numerical simulation of the experimental spectra. Experimental conditions: microwaves, 2 mW at 9.65 GHz; temperature, 19 K. Numerical simulations of the substrate-free nitrosyl complex have been omitted because the electronic parameters obtained from these simulations are the same as that of the histidine-bound nitrosyl complex due to their nearly identical EPR spectra.

Similar EPR experiments were also performed on the GDO. However, the ferrous center in the GDO is highly susceptible to oxidation. In addition to this, in our preparations, recombinant GDO consistently exhibits low iron occupancy. Due to this, the EPR spectra of GDO nitrosyl complexes exhibited poor signal-to-noise ratios (Figure S1), preventing their detailed analysis. However, similar to SDO, the EPR spectra of Fe­(II)-NO and Fe­(II)-His-NO complexes of GDO are identical and binding of gentisate introduces distortion to the metal center in the gentisate-bound nitrosyl complex. Taken together, these results provide two key findings. First, binding of histidine to SDO and GDO does not obstruct O_2_ binding, and the observed inhibition of the reaction does not originate from the lack of O_2_ access at the catalytic site. Second, the binding modes of histidine and the substrate (gentisate) to the catalytic cavity are not identical.

### Inhibitor Binding Mode

Lack of detectable inhibition by small amino acids such as glycine and alanine suggests that metal-binding groups afforded by the backbone amino and carboxylate moieties alone are not sufficient for binding to the iron core to inhibit the reaction. Instead, the side chain groups of the inhibitory amino acids may be critical for inhibitor binding. All three inhibitory amino acids bear polar or charged side chains, suggesting that their binding may be assisted by electrostatic interactions with the active site cavity. While GDO has been crystallographically characterized in its substrate-free form, structures of the substrate-bound form are not available. In contrast, various substrate-bound structures of SDO have been reported, which demonstrate significant structural rearrangement upon substrate binding. Being a member of the GDO family, SDO and GDO share high sequence similarity (up to ∼70%) and bear overall comparable substrate-free structures. Considering this and given that SDO has been structurally characterized in greater detail compared to GDO, below we provide an analysis of possible inhibitor binding modes by focusing on the crystal structures of SDO and suggest that our conclusion also extend to the general GDO family.

The crystal structure of SDO (PDB: 3NJZ) shows that the active site cavity surface in SDO is primarily electrostatic with several accessible polar and charged amino acids. For instance, the catalytic pocket is composed of residues such as R83, W104, R127, H162, Q168, and D174. Therefore, the catalytic pocket may support inhibitor binding via electrostatic interactions in addition to the expected hydrophobic contributions. Given histidine exhibits the strongest inhibition (*K*_d_ = 80 μM), we further evaluated the origin of its binding to the catalytic center. The lack of attenuation of reaction kinetics by glycine and alanine shows that the side chain imidazole moiety of histidine is critical for binding. To evaluate the contributions of the side chain, we monitored the influence of imidazole on reaction kinetics. Steady-state kinetics measurements in the presence of imidazole and performed under similar conditions as other amino acids did not show any inhibition suggesting that histidine side chain alone cannot regulate binding. Combined, these results demonstrate that both side chain and backbone functional groups together enable histidine binding. Furthermore, the chirality of the molecule also plays a role. Similar to imidazole, no attenuation of the reaction kinetics was observed for the reactions performed in the presence of d-histidine (Table 1).

For the observed competitive inhibition to take place, inhibitory amino acids may either occupy the substrate-binding site or impede the access of the substrate to its binding site. On the basis of the crystal structure, substrate binding to SDO takes place via a bidentate mode in which the carboxylate and the cis hydroxyl groups of the substrate ligate to the ferrous center. The EPR studies reported here show that binding of histidine at the catalytic cavity does not introduce distortions at the metal center similar to that imparted upon the bidentate substrate ligation, thereby suggesting an alternate binding mode. To further evaluate the binding modes of inhibitory amino acids, we performed docking studies modeling their interactions in the catalytic center. Docking studies do not fully account for ligand flexibility and are based on simplistic force fields and may yield unphysical binding configurations. To test their reliability, we first performed docking calculations on the natural substrates salicylate and gentisate. In the lowest-energy structure generated during the docking calculations, the substrate molecule ligates to the ferrous center in a bidentate binding mode supported by its carboxylate and hydroxyl groups (Figures 3a and S2). This binding mode is in good agreement with the reported substrate-bound crystal structures (Figure 3b,c)^19^ and demonstrates the feasibility of docking calculations in predicting inhibitor binding configurations. Despite this, we recognize the inherent limitations of these calculations in predicting the accurate inhibitor binding modes and energies. Therefore, in the following discussion, the results of docking calculations have been only used to gain insights into the potential interactions favoring the inhibitor binding within the active site. However, additional binding configurations not predicted by the docking calculations may exist.

**Figure 3:**
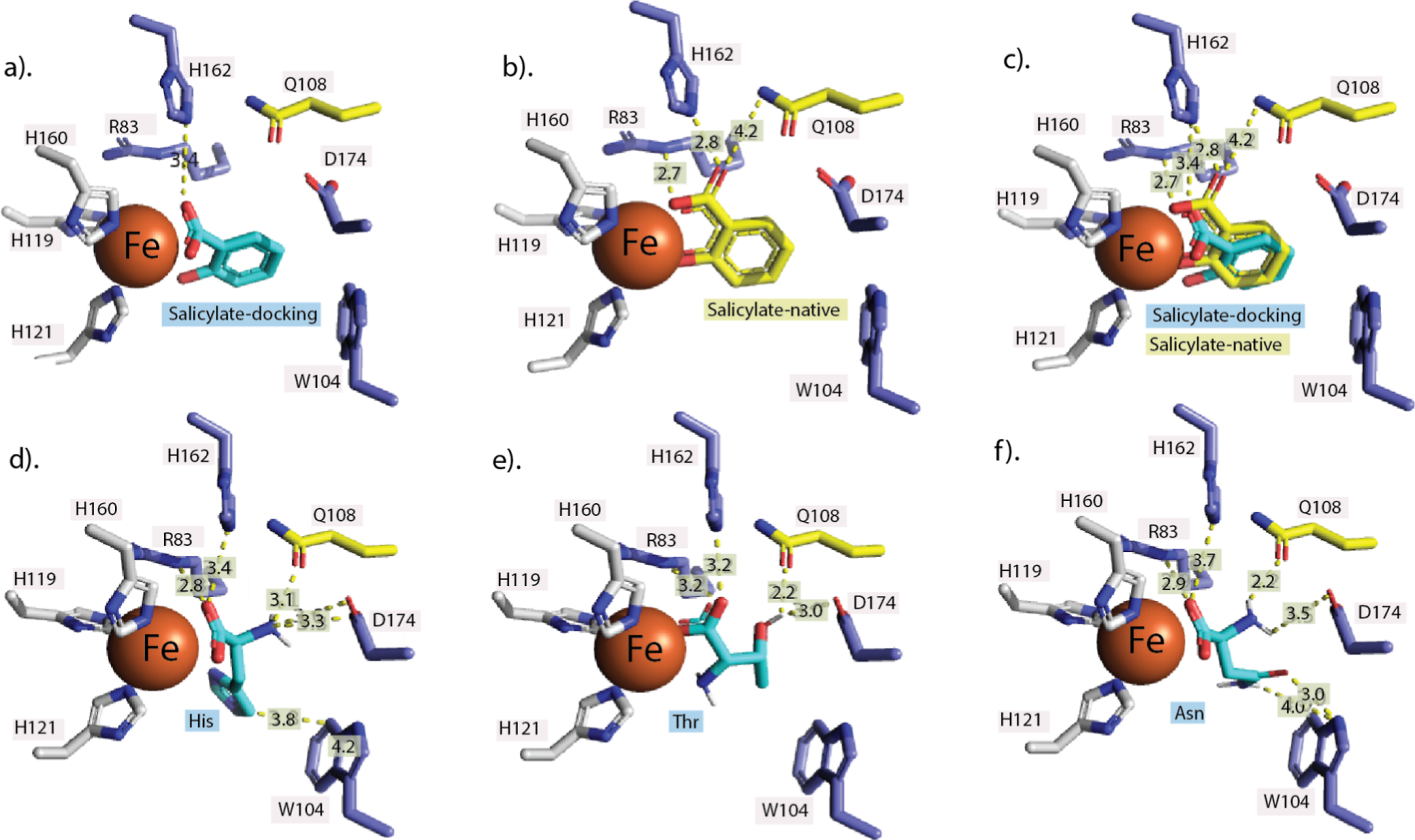
Binding modes of substrates and inhibitory amino acids to the catalytic cavity of SDO based on docking studies. (a) Predicted binding mode of salicylate; (b) crystal structure of salicylate bound to SDO (PBD: 3NJZ); (c) overlay of the crystal structure and the lowest energy docking structure of salicylate. (d–f) Predicted lowest energy structures of histidine, threonine, and asparagine, respectively.

The structures of the lowest energy configurations obtained from docking of the inhibitors into the catalytic site show a conserved orientation among all molecules and provide a molecular basis for their binding to the catalytic cavity. The corresponding docking scores for the lowest energy configurations are reported in Table S1. As shown in Figure 3d,f, the backbone carboxylate groups of histidine and asparagine interact with the iron center, while their amino group points toward D174 and Q108, except for threonine. This binding mode of the inhibitors via the carboxylate group is monodentate as opposed to the bidentate binding of the natural substrate. The average distance between the amino group of inhibitors and carboxylate oxygens of D174 or Q108 is ∼3 Å, suggesting that the binding mode is stabilized via electrostatic interactions. These interactions force the amino acid inhibitor side chains to make contact with residues such as W104. In addition to this, other residues such as A125, L176, I178, and F189 may promote hydrogen bonding or hydrophobic interactions. It is the interactions of the inhibitor side chain with W104 and other neighboring residues that may dictate its binding as described below.

In the lowest energy docked structure of l-histidine, the imidazole moiety is within 4–5 Å of the side chain of residue W104 and ∼3 Å away from D174 (Figure 3d). Furthermore, while the side chain imidazole moieties of histidine ligands directly tether to the ferrous ion in the catalytic pocket, docking calculations suggest that the imidazole ring of free histidine does not directly bind to the metal center. These docking results are in agreement with the experimental findings demonstrating that imidazole itself does not attenuate the reaction kinetics. In contrast to l-histidine, the chirality of d-histidine may force its side chain to point away from residues capable of making stable contacts (Figure S3) needed for binding to the catalytic cavity, thereby rendering it incapable of attenuating the reaction kinetics.

The binding of threonine may be stabilized by the interaction of the methyl group with A125, L176, and I178 of the protein polypeptide. Support for this argument comes from no observed inhibition by serine, which bears the hydroxyl and the backbone carboxylate moieties but lacks the side chain methyl group. Collectively, this analysis suggests that while the backbone carboxylate moiety of the inhibitor interacts with the ferrous center, favorable interactions of the side chain groups with the residues in the catalytic cavity are key for stable binding. Our EPR studies demonstrate that inhibitor binding does not alter the symmetry of the metal center based on similar E/D values obtained for the ferrous-nitrosyl complexes with and without an inhibitor. In the context of the discussion presented above, our results suggest that compared with the bidentate substrate binding, which distorts the geometry of the metal center, the monodentate ligation of the inhibitor molecule to the metal center does not alter its geometry but blocks substrate access.

### Putative Relevance of Amino Acid Inhibition of the GDO/SDO Reaction

The results presented herein demonstrate for the first time amino acid-assisted inhibition of aromatic ring-cleaving dioxygenases. Given the critical role of aromatic ring-degrading enzymes in sustaining the global carbon cycle^8^ and the prevalence of free amino acids in the cell,^32,33^ our findings may suggest that the availability of free amino acids could be utilized in nature to regulate aromatic compound degradation by microorganisms. Based on our results, for GDO and SDO, this inhibition is primarily controlled by histidine. Below, we provide a hypothesis describing cellular regulation of aromatic ring degradation by histidine. We remark that while our current work does not provide evidence for the biological existence of such amino-acid-mediated regulation, the following discussion is intended to provide a basis for future work.

Aerobic degradation of aromatic compounds in nature takes place through one of four intermediates: (i) catechol, (ii) protocatechuate, (iii) gentisate, or (iv) hydroquinone.^8^ Products of the gentisic acid pathway enter the TCA cycle as pyruvate and fumarate. SDO and GDO participate in this pathway, and inhibition of their reaction by selected amino acids in cellular conditions will result in its deactivation. Histidine, which is synthesized via the pentose phosphate pathway, may indirectly regulate GDO/SDO activity. When glucose is abundant, activation of glycolysis and the pentose phosphate pathway will result in increased cellular histidine concentration, thereby potentially attenuating the gentisic pathway upon GDO inhibition. Conversely, under glucose-limited conditions when the pentose phosphate pathway is suppressed, activation of the gentisic pathway may take place due to decrease in histidine synthesis. Under these conditions, while the supply of pyruvate from glycolysis is limited, the gentisic pathway could continue to feed the TCA cycle (with pyruvate and fumarate) for cellular metabolism (Scheme 1). It is noteworthy that the efficiency of energy production via glycolysis far exceeds that of the gentisic pathway. During glycolysis, 30–32 ATP molecules are produced for each glucose molecule consumed, resulting in an effective generation of ∼5 ATP per oxygen atom.^34^ In contrast, the gentisic pathway only generates 2.5 ATP per oxygen atom, suggesting that glycolysis-driven metabolism is expected to be the primary mechanism of energy generation in cells.

**Scheme 1:**
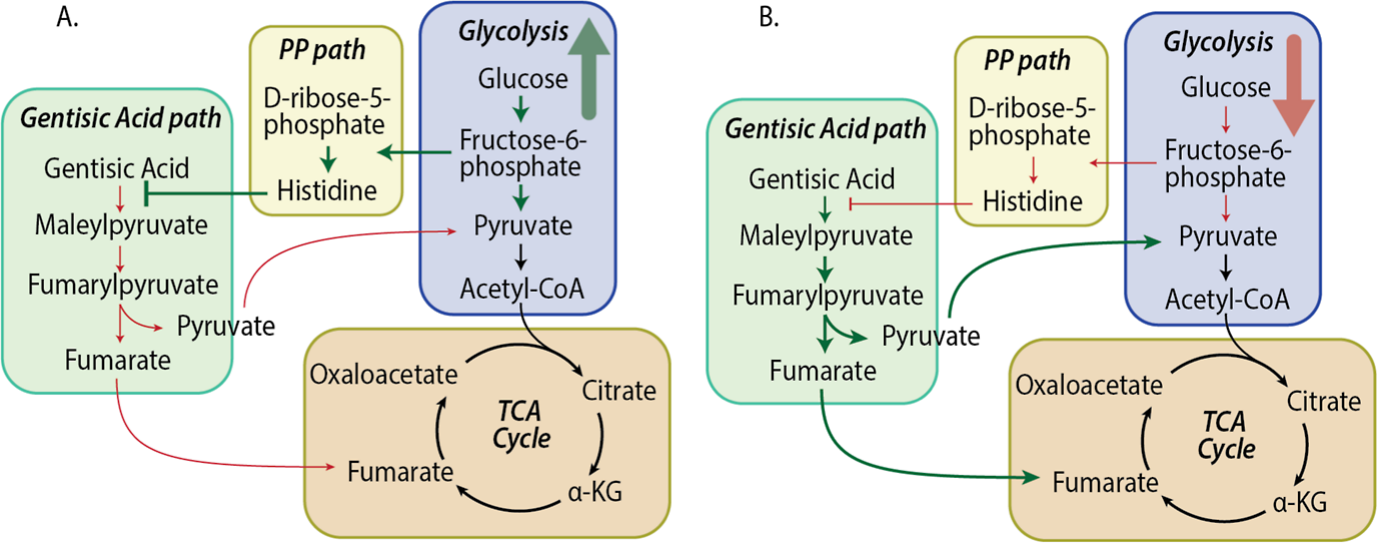
Proposed Regulation of the Gentisic Pathway by Histidine under High (A) and Low (B) Glucose Conditions

## Conclusions

In this report, we demonstrate that aromatic ring-degrading dioxygenases are inhibited by natural amino acids. While most amino acids bear metal-ligating carboxylate and amino groups, which may inhibit the chemical reaction by competing with substrate binding, only histidine strongly hinders the catalysis by GDO and SDO. In addition to this, threonine and asparagine are mild inhibitors (on the basis of measured *K*_d_ values) of SDO but not GDO. These results demonstrate the importance of active-site interactions in modulating enzymatic functions. EPR studies demonstrate that inhibitor binding to the catalytic cavity does not hinder the access of the cosubstrate, O_2_. In addition, unlike substrate binding, inhibitor binding does not alter the geometry of the metal center. Considering that histidine is synthesized by the pentose phosphate pathway and based on our results, we propose a feedback loop that may utilize this observed inhibition of the gentisic pathway by natural amino acids to regulate cellular energy production while modulating the concentration of free amino acids in the cell. Our findings may extend to other ring-cleaving oxygenases, particularly catechol dioxygenases that participate in aromatic compound degradation via catechol and protocatechuate intermediates. The results presented herein may provide a basis for future studies evaluating cellular regulation of aromatic compound degradation pathways.

## Supplementary Material



## Notes

EPR spectra of ferrous-nitrosyl complexes of GDO in the presence and absence of histidine; binding orientation of gentisate in the catalytic cavity of SDO from docking studies; comparison of l- and d-histidine docking structures; and predicted inhibitor binding affinities (PDF)
