# Supplementary material for: Inhibition of Ring-Cleaving Dioxygenases by Natural Amino Acids
Source: ACS Omega. 2026 Jun 1;11(23):34659–67. doi: 10.1021/acsomega.6c03268 (PMC13280823; doi:10.1021/acsomega.6c03268)
Supplement: Supplementary file 1 [file ao6c03268_si_001.pdf]

Supporting Information for

Inhibition of Ring Cleaving Dioxygenases by Natural Amino Acids

Qian Wang[‡,#], Hanbin Li[‡,#] and Rupal Gupta[‡,⊥,*]

[‡]*Department of Chemistry, College of Staten Island, City University of New York, New York, 10314, United States*

[⊥]*Ph.D. Programs in Biochemistry and Chemistry, The Graduate Center of the City University of New York, New York, 10016, United States*

[#] These authors contributed equally

***Corresponding author:** Rupal Gupta, Department of Chemistry, College of Staten Island, The City University of New York, USA, email: rupal.gupta@csi.cuny.edu; Qian Wang, Department of Chemistry, College of Staten Island, The City University of New York, USA, email: qian.wang@csi.cuny.edu

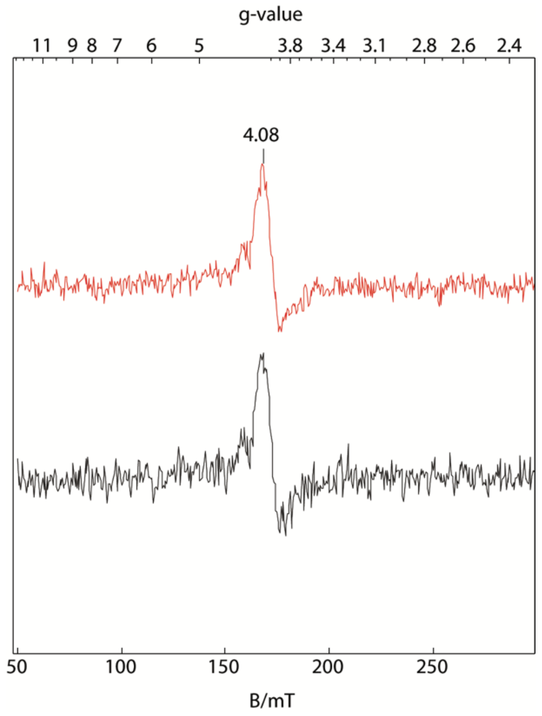

Figure S1. EPR spectra showing the resonance near g=4 region of ferrous nitrosyl complexes of GDO in the absence a) and presence b) of histidine. Experimental conditions: microwaves, 2 mW at 9.65 GHz; temperature, 19 K.

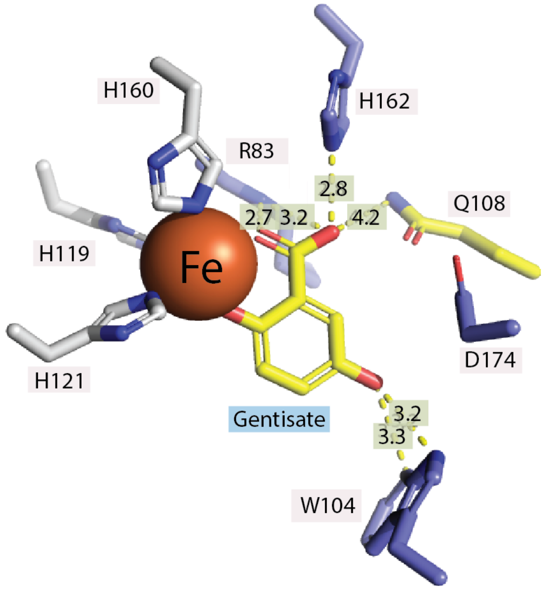

Figure S2. Binding orientation of gentisate in the catalytic cavity of SDO obtained from docking studies.

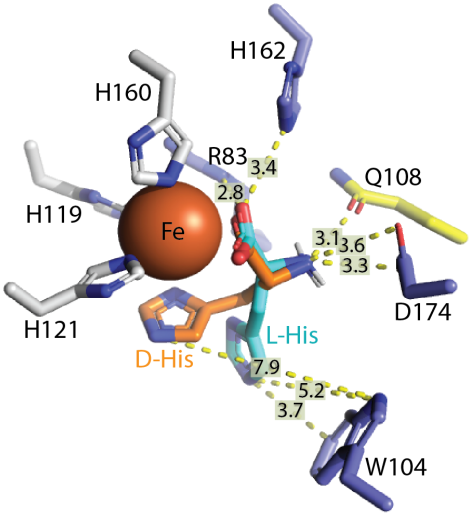

Figure S3. A comparison of the lowest energy structures obtained from docking calculation of L- (yellow) and D-histidine (cyan) in SDO catalytic cavity.

Table S1. The results of predicting binding affinity of the potential inhibitors of SDO by AutoDock.

**Amino Acids**	**Affinity (kcal/mol)**
Salicylic Acid	-6.8
L-histidine	-5.5
L-threonine	-5.0
L-asparagine	-5.3
D-histidine	-5.4
L-serine	-4.4
L-cysteine	-4.4
Imidazole	-3.3
